# Maternal Mortality Among Black Women in Brazil: A Retrospective Cohort Study

**DOI:** 10.3390/ijerph23010094

**Published:** 2026-01-09

**Authors:** Gustavo Gonçalves dos Santos, Anuli Njoku, Reginaldo Roque Mafetoni, Clara Fróes de Oliveira Sanfelice, Ana Izabel Oliveira Nicolau, Patrícia Wottrich Parenti, Cely de Oliveira, Leticia López-Pedraza, Ricardo José Oliveira Mouta, Karina Franco Zihlmann, Cindy Ferreira Lima, Cícero Ricarte Beserra Júnior, Cláudia de Azevedo Aguiar, Cesar Henrique Rodrigues Reis, Júlia Maria das Neves Carvalho, Ana Cristina Ribeiro da Fonseca Dias, Maria Luísa Santos Bettencourt, Mónica Alexandra Pinho da Silva, Maria João Jacinto Guerra, Giovana Aparecida Gonçalves Vidotti

**Affiliations:** 1 Escola Paulista de Medicina, Universidade Federal de São Paulo (EPM/UNIFESP), São Paulo 04023-062, SP, Brazil; goncalves.giovana2@gmail.com; 2 Campus Guarujá (UNAERP), Universidade de Ribeirão Preto, São Paulo 11440-003, SP, Brazil; celyoliveira@unaerp.br; 3 Programa de Pós-graduação do Departamento de Ginecologia, Edifício Octávio de Carvalho, Rua Botucatu, 740-5º andar, Sala 508, São Paulo 04023-062, SP, Brazil; 4 Department of Public Health, College of Health and Human Services, Southern Connecticut State University (USCS), New Haven, CT 06515, USA; njokua3@southernct.edu; 5 Faculdade de Enfermagem, Universidade Estadual de Campinas (UNICAMP), Campinas 13083-887, SP, Brazil; mafetoni@unicamp.br (R.R.M.); clarafos@unicamp.br (C.F.d.O.S.); 6 Centro de Ciências da Saúde, Departamento de Enfermagem, Universidade Federal do Ceará (UFC), Fortaleza 60430-160, CE, Brazil; anaizabelnicolau@gmail.com; 7 Escola de Artes, Ciências e Humanidades, Universidade de São Paulo (EACH/USP), São Paulo 03828-000, SP, Brazil; pwparenti@usp.br; 8 Facultad de Enfermería, Fisioterapia y Podología, Universidad Complutense de Madrid (UCM), 28040 Madrid, MA, Spain; letilo03@ucm.es; 9 Faculdade de Enfermagem, Universidade do Estado do Rio de Janeiro (UERJ), Rio de Janeiro 20551-030, RJ, Brazil; ricardomouta@hotmail.com; 10 Departamento de Saúde, Educação e Sociedade, Universidade Federal de São Paulo-Campus Baixada Santista (UNIFESP/BS), São Paulo 11060-001, SP, Brazil; karina.zihlmann@unifesp.br; 11 Organização Pan-Americana da Saúde (OPAS), Secretaria do Estado de Saúde de Minas Gerais, Minas Gerais 31630-903, MG, Brazil; cindy.lima@alumni.usp.br; 12 Estratégia Saúde da Família do Município de Senador Pompeu (ESF), Pompeu 63600-000, CE, Brazil; ricartebeserra.enfermeiro@gmail.com; 13 Instituto de Ciências da Saúde, Universidade Federal do Triângulo Mineiro (UFTM/ICS), Uberaba 38025-180, MG, Brazil; claudia.aguiar@uftm.edu.br; 14 Programa de Pós-graduação em Enfermagem, Faculdade de Enfermagem, Universidade Federal de Mato Grosso do Sul (UFMS), Três Lagoas 79070-900, MS, Brazil; cesar.h@ufms.br; 15 Unidade Científico-Pedagógica de Enfermagem de Saúde Materna, Obstétrica e Ginecológica (UCP-ESMOG), Escola Superior de Enfermagem de Coimbra (ESEnfC), 3046-851 Coimbra, Portugal; juliacarvalho@esenfc.pt; 16 Escola Superior de Saúde, Universidade dos Açores (UAc/ESS), 9500-315 Ponta Delgada, Portugal; anacrfdias@gmail.com (A.C.R.d.F.D.); maria.ls.bettencourt@uac.pt (M.L.S.B.); 17 Escola Superior de Enfermagem do Porto (ESEP), Universidade do Porto, 4200-072 Porto, Portugal; monicasilva@esenf.pt (M.A.P.d.S.); mjguerra@esenf.pt (M.J.J.G.)

**Keywords:** women’s health, maternal mortality, structural racism, health inequalities, health equity

## Abstract

Background: Maternal mortality in Brazil remains a critical indicator of social and racial inequalities, reflecting structural failures in access to and quality of obstetric care. Black women, particularly those categorized as black or brown, are at a higher risk of dying during pregnancy, childbirth, or the postpartum period. This is the result of the intersection of institutional racism, poverty, and social vulnerabilities. This study aimed to analyze trends and associated factors of maternal mortality among black women in Brazil from 2000 to 2020. Methods: This is a retrospective cohort analytical study using data from the Brazilian Mortality Information System. The sample included women aged 10 to 49 years whose underlying cause of death was classified under ICD-10 codes O00–O99. Descriptive and bivariate analyses were conducted, as well as Poisson and multinomial logistic regressions to estimate adjusted risk ratios according to skin color, education, region, type, and place of death. Results: A total of 40,907 maternal deaths were identified, with 59.2% occurring among black women. The maternal mortality ratio was 39% higher among black women compared to white women and more than double among Indigenous women. Low education, residence in the North and Northeast regions, deaths outside hospital settings, and lack of formal investigation were independently associated with increased risk. Direct obstetric causes accounted for most deaths, with hypertensive disorders and puerperal complications being the leading conditions. Conclusions: Maternal mortality among black women in Brazil reveals deep structural inequalities. Urgent public policies that incorporate an intersectional perspective, addressing race, gender, and class, are necessary to reduce disparities and ensure equitable and dignified maternal healthcare.

## 1. Introduction

Maternal mortality, defined as the death of a woman during pregnancy or in the 42 days following the end of gestation, remains one of the most alarming indicators of health inequality in Brazil. Among the various population groups affected, black women face higher risks of death during pregnancy, childbirth, and the postpartum period. The intersection of structural racism, social vulnerability, and inequalities in obstetric care puts this population at a disadvantage in terms of reproductive rights and access to comprehensive health. Institutionalized racism in the health system contributes to negligence, delays in care, and underreporting of maternal complications, which directly impacts obstetric outcomes for black women [1].

In Brazil, the Maternal Mortality Ratio (MMR) remains high at around 54.7 deaths per 100,000 live births in 2021, according to the Ministry of Health, and shows marked racial and regional disparities. Healthcare is largely provided by medical professionals, in a highly medicalized and interventionist model, with high rates of cesarean sections compared to vaginal births, resulting in a higher incidence of complications and harmful outcomes for women. Black women face a significantly higher risk of death during pregnancy, childbirth, and the postpartum period, due to the intersection of structural racism, social vulnerability, and inequality in obstetric care, which highlights the persistence of barriers to access to qualified and safe care [1,2]. A study conducted in the state of Rio de Janeiro (2008–2021) confirms that, although there has been an overall reduction in the MMR, the differences between white and black women remain significant, with the risk of death among black women doubling in some years [2].

A qualitative study indicated that this disparity reflects the absence of effective public policies aimed at racial equity in health and the perpetuation of institutional racism, which manifests itself in negligent care, delays in transfers, and underreporting of obstetric complications. The care provided to black women is frequently marked by discrediting their complaints, minimizing pain, and a lack of empathetic listening, compromising the quality of care and worsening potentially avoidable outcomes [3].

The social determinants of health, such as income, education, housing, and labor market participation, amplify this vulnerability, especially in the poorest and most peripheral regions, where prenatal and obstetric emergency care is insufficient [4]. An emblematic example of this scenario is the case of Alyne da Silva Pimentel, a young black woman living in the Baixada Fluminense, who died in 2002 after negligent care. Recognized by the Commission on the Elimination of Discrimination against Women (CEDAW), the case exposed the responsibility of the Brazilian State for violating reproductive human rights and revealed how the naturalization of racial inequalities structures the health system [5,6].

Although the difficulties of analysis are aggravated by the underreporting of the race variable, Brazilian studies confirm the increased risk of maternal death among black women, with rates two to eight times higher in states such as Paraná and Rio de Janeiro [7]. This reality is part of a global context of racial inequities in maternal mortality. In the United States, non-Hispanic black women have a two to three times higher risk of dying from pregnancy-related causes than non-Hispanic white women, with a rate of 69.9 per 100,000 live births compared to 26.6 among white women [8,9]. Between 2000 and 2019, more than 21,000 women died during pregnancy or in the postpartum period, with black and indigenous women being the most affected [10,11].

The COVID-19 pandemic further exacerbated this inequality: in March 2022, black women accounted for approximately 54% of COVID deaths, in addition to representing almost half of admissions to Intensive Care Units (ICUs) [12]. The literature indicates that these disparities persist even in the face of medical advances, driven by structural racism, unequal access, and low cultural competence of healthcare teams [12,13].

In Brazil, the organization of the health system plays a decisive role in shaping maternal outcomes. The Unified Health System (Sistema Único de Saúde—SUS), established by the 1988 Federal Constitution, guarantees universal and free access to healthcare and is responsible for the majority of births in the country, particularly among socially and economically vulnerable populations. Despite significant progress in expanding coverage and surveillance of maternal deaths, major gaps persist in the quality of care, resource distribution, and the integration between primary care and specialized obstetric services [1,2,3].

The Brazilian obstetric care model remains predominantly medicalized and hospital-centered, with more than 98% of births occurring in hospital settings and the majority attended by physicians. Cesarean sections are notably frequent, accounting for more than half of all deliveries, with rates exceeding 70% in the private sector and around 45% in the public SUS network [10,11]. Although the expansion of hospital-based care has contributed to the reduction in maternal deaths over the last two decades, excessive medical interventions, limited midwife participation, and regional inequalities in access to high-quality obstetric services continue to influence adverse outcomes [12,13].

In this context, the Rede Alyne is a program of the Ministry of Health that aims to promote the monitoring and improvement of maternal and child health services, through the association of strategies that link professional training, improvement of service management, and continuous analysis of epidemiological data, in order to strengthen the surveillance of maternal and child mortality in Brazil, aligned with the Sustainable Development Goal (SDGs), especially number 3, which is directly aimed at reducing maternal mortality worldwide. Thus, an epidemiological study that explores Brazilian maternal mortality data in connection with the potential of the Rede Alyne and the commitments assumed by the SDGs could provide support for the formulation of more effective public policies, aimed at equity and quality in obstetric care.

Thus, the question is: What are the sociodemographic, clinical and contextual factors associated with the increased risk of maternal mortality among black women in Brazil between 2000 and 2020? Aiming to analyze the sociodemographic, clinical and territorial factors associated with maternal mortality among black women in Brazil in the period from 2000 to 2020, with an emphasis on racial and regional inequalities.

## 2. Materials and Methods

### 2.1. Study Design

This is a retrospective cohort analytical observational study

### 2.2. Study Population and Source of Information

Data extracted from the Sistema de Informações sobre Mortalidade (SIM), through the public platform Tabulador de Dados da Internet of the Departamento de Informação e Informática do Sistema Único de Saúde (TABNET/DATASUS). All female deaths that occurred in Brazil, aged between 10 and 49 years (operational definition of childbearing age according to the Ministry of Health), whose underlying cause of death was classified in codes O00 to O99 of the International Classification of Diseases—10th Revision (ICD-10), corresponding to direct or indirect maternal death, during the period of interest were included. Death records that met the following criteria were included: (a) pregnant and postpartum women; (b) underlying cause of death related to pregnancy, childbirth or puerperium (ICD-10: O00–O99); (c) occurrence between 1 January 2000 and 31 December 2020; and (d) information completed for the skin color field. Records with inconsistent data were excluded.

The final sample comprised 40.907 maternal deaths nationwide between 2000 and 2020, with a predominance of black women (black and brown women representing approximately 65% of the sample). With this sample size, the study presented a statistical power greater than 95% to detect differences in MMR between racial groups with a relative risk ≥ 1.2, assuming an alpha error of 5% and an exposure ratio of 2:1.

The study period from 2000 to 2020 was selected for three main reasons. First, 2000 marks the beginning of the consistent availability of national data on maternal deaths with standardized classification according to ICD-10 in the SIM/DATASUS, allowing longitudinal and comparable analyses across all Brazilian regions. Second, this period coincides with the implementation of major maternal health policies and surveillance programs in Brazil, such as the Comitês de Mortalidade Materna and the Rede Cegonha, enabling the evaluation of trends before and after the adoption of these initiatives. Third, extending the analysis to 2020 allows the inclusion of recent data encompassing the COVID-19 pandemic period, which had a notable impact on maternal outcomes and deepened existing racial and regional inequalities. Therefore, the 2000–2020 timeframe ensures both historical depth and contemporary relevance for understanding long-term patterns and persistent inequities in maternal mortality among black women in Brazil.

### 2.3. Study Variables

The variables used in this study were extracted from the SIM/DATASUS database, publicly available on the TABNET platform. The database includes records of maternal deaths coded according to the ICD-10, in the code range O00 to O99, which cover causes related to pregnancy, childbirth and puerperium. The main exposure variable was the woman’s race, categorized according to the standards of the Instituto Brasileiro de Geografia e Estatística (IBGE): white, black, mixed race, Asian and indigenous. For the analyses, an aggregate category of black women (black and mixed race) was created, as opposed to the others, with the aim of highlighting racial inequalities in maternal mortality.

Although the study does not include a direct or self-reported measure of racism, racial inequalities were examined using the variable “race”, as recorded in the official death certificates of the SIM/DATASUS. This variable follows the classification of the IBGE: White, Black, Brown (mixed race), Indigenous, and Asian and allows for the identification of disparities in maternal mortality rates between racial groups. These disparities are interpreted in light of the concept of structural racism, understood as a set of social, institutional, and historical mechanisms that systematically disadvantage Black and Indigenous populations in access to health, education, income, and territorial resources.

Therefore, rather than attempting to quantify racism as an individual-level exposure, the analysis adopts a population-based and structural perspective, assessing racial inequities as expressions of broader social and institutional processes that shape maternal health outcomes. This interpretative approach is consistent with previous epidemiological studies that use racial disparities in health indicators as indirect evidence of structural and institutional racism in healthcare systems [14,15,16,17,18].

The outcome variable was maternal mortality, defined as the number of deaths recorded in women with underlying causes related to pregnancy, childbirth or puerperium. The MMR was calculated, expressed as the number of maternal deaths per 100,000 live births, using data from the Sistema de Informações sobre Nascidos Vivos (SINASC) as the denominator. Demographic variables were also considered, such as the woman’s age at the time of death, recorded in age groups, and the region of residence (North, Northeast, Southeast, South and Central-West), which allowed the identification of regional risk patterns.

Among the socioeconomic and welfare variables, the woman’s education level was analyzed, categorized by years of study (none, 1–3 years, 4–7 years, 8–11 years, 12 years or more, unknown), and marital status (single, married or in a stable union, separated, widowed or unknown), used as proxies for social vulnerability.

The variable “place of death” was considered to identify whether the death occurred in a health facility, home, public road or other location, which is essential for investigating access failures and timely response from the health system. The variable “gestation time” was also used, which indicates whether the death occurred during pregnancy, childbirth or puerperium (up to 42 days after childbirth), and is important for differentiating types of direct or indirect maternal mortality.

Finally, the variable “year of death” was essential for temporal analyses and for identifying trends or changes in the pattern of maternal mortality over the years. This variable allowed, for example, a comparison between pre-pandemic and COVID-19 pandemic periods, providing support for assessing the resilience and weaknesses of the health system in the face of health crises. The combination of these variables allowed descriptive and inferential analyses, ensuring comparability between racial and regional groups, as well as adjustment for potential confounding factors.

### 2.4. Statistical Analysis

Initially, a univariate descriptive analysis was performed to characterize the population according to sociodemographic, obstetric, and regional variables, with absolute and relative frequencies. Then, a bivariate analysis was conducted with calculation of the MMR stratified by skin color, year, age group, and region. To estimate the magnitude of the association between skin color and maternal mortality, Poisson regression models were used, adjusted for age, education, and region. The results were expressed as relative risk ratios (RR) with respective 95% confidence intervals (95% CI). Statistical analysis was performed using Stata version 17.0 software (StataCorp, College Station, TX, USA).

Contingency tables were prepared by cross-referencing the variables to analyze the distribution of maternal deaths according to skin color, education, region, type of death, and other clinical characteristics. To verify the existence of a statistical association between categorical variables, chi-square (χ^2^) tests were performed, considering statistical significance for *p* < 0.05. These tests allowed the identification of regional and social inequalities, especially highlighting the disparities in the risk of maternal death between racial groups and levels of education.

To estimate the factors associated with the risk of maternal mortality, Poisson regression with robust variance was used, including the logarithm of the number of live births per stratum as an offset, in order to control the effect of the population base. Independent variables such as skin color, education, region, type of death, ICD-10 chapter and group, place of death, and investigation status were included in the model. This model allowed the calculation of adjusted risk ratios (RRadj), confidence intervals (95% CI), and *p*-values, highlighting the independent factors associated with increased or reduced risk.

The study acknowledged the possibility of underreporting or misclassification of the underlying cause of death, particularly in regions with incomplete SIM coverage or precarious health services. Furthermore, it considered possible differential classification bias, given the non-standardized filling of the skin color variable in the certificates.

### 2.5. Ethical Aspects

This study used exclusively anonymized public domain secondary data, extracted from an open platform of the Ministry of Health, and it was not possible to individually identify the subjects. In accordance with Resolution No. 510/2016 of the National Health Council, which regulates research with public data, submission to the Research Ethics Committee was not necessary.

## 3. Results

As shown in Table 1, the highest proportion of maternal deaths occurred in the 20–29 age group (40.05%), followed by 30–39 years (38%). Adolescents aged 15–19 years represented 12.48% of the total, highlighting the relevance of maternal mortality at early ages. Deaths in girls aged 10–14 years, although less frequent, totaled 346 cases (0.85%), which is epidemiologically relevant, considering that pregnancy in this age group is high risk. Cases outside the standard reproductive range (≥50 years) are rare and may reflect recording errors or exceptional situations (such as pregnancy in induced menopause).

Brown women accounted for the largest proportion of maternal deaths, with 48.33% of the total, followed by white women (33.37%) and black women (10.87%). When aggregated, the black and brown categories total 24,219 deaths, representing 59.20% of the total, that is, the majority of maternal deaths occurred among black women, reinforcing racial inequalities. The indigenous category accounted for 1.38% of cases, a significant value when compared to its population proportion. The number of records with unknown race (5.75%) is relatively low, allowing robust analyses based on this variable. The yellow category represented only 0.30%, which may limit specific statistical analyses in this group.

The Southeast region accounts for the highest proportion of maternal deaths (34.55%), closely followed by the Northeast region (33.61%). The North (12.58%) and South (11.14%) regions had intermediate proportions. The Central-West region, although with a smaller absolute population, accounted for 8.13% of deaths. When adjusted for the number of live births, these values may reflect regional inequality in access to and quality of obstetric care, especially in the North and Northeast regions, historically marked by greater socioeconomic vulnerability and lower coverage of specialized services.

More than half of the women who died from maternal causes were single (50.30%), which may reflect situations of greater social vulnerability, less family support and possible limited access to health services. Married women or women in stable unions accounted for approximately 30.17% of cases, suggesting that marital status does not necessarily protect against maternal mortality. The categories “other” (possibly consensual, unspecified or unstable union) and “legally separated” together account for almost 11%, reinforcing the diversity of family arrangements. The presence of missing data (7.74%) is moderate, but should be considered in the analyses to avoid information bias.

Most maternal deaths occurred among women with 8 to 11 years of schooling (29.69%), followed by those with 4 to 7 years (23.33%) and 1 to 3 years (11.39%), indicating that more than half of the deceased women had completed primary education at most. Only 9.11% of the women had completed 12 or more years of schooling, which represents a minority with completed secondary education or higher. The proportion of women with no schooling is 3.75%, a number that is still significant in absolute terms. The percentage of unknown schooling (22.74%) is high, which may compromise analyses adjusted for this factor and requires caution in interpreting the results.

As shown in Table 2, although the focus of maternal deaths is on direct obstetric causes (O00–O99), approximately 2.18% of deaths occurred due to indirect causes linked to other clinical conditions aggravated by pregnancy. Most cases of indirect causes are in Chapter I—infectious and parasitic diseases (2.06%), which may include cases of HIV, non-obstetric sepsis and tuberculosis. Mental disorders (0.09%) and neoplasms (0.02%) have a very small share, but indicate the presence of psychiatric and oncological conditions interfering with the course of pregnancy. Death due to endocrine causes was recorded only once, which may be related to poor control of diseases such as diabetes or thyroid disorders.

The most frequent group was “other obstetric conditions not classified elsewhere” (29.98%), which includes poorly defined causes or deaths with little specific coding, which may indicate failures in the certification of the cause of death. In second place, hypertensive disorders of pregnancy, childbirth and puerperium stand out with 21.39%, one of the main direct obstetric causes of maternal death in Brazil. Complications of labor and delivery (15.23%) and complications related to the puerperium (13.26%) also represent significant causes, reinforcing the importance of qualified hospital obstetric care. Abortion (7.81%) as the underlying cause continues to be a public health problem, with emphasis on the risks associated with unsafe abortion, especially among black and low-income women. Indirect causes (such as HIV, mental disorders and neoplasms) combined account for approximately 2.18% of deaths, with HIV being the most significant (2.06%).

Direct obstetric deaths caused by obstetric complications during pregnancy, childbirth or the puerperium (such as hemorrhages, infections, hypertensive disorders, uterine rupture, etc.) accounted for 65.93% of the total. This number reinforces that most maternal deaths could be avoided with qualified, timely and accessible obstetric care. Indirect obstetric deaths, which result from pre-existing clinical conditions or those acquired during pregnancy that worsened during pregnancy (such as HIV, heart disease, diabetes, neoplasms), accounted for 30.96%. This high percentage highlights the importance of preconception care and comprehensive assistance to women’s health, in addition to greater coordination between primary and specialized care. Unspecified deaths accounted for 3.13%, a relatively low figure, but which still indicates limitations in filling out the death certificate, especially in the fields of underlying cause and relationship to pregnancy, which hinders correct classification and epidemiological surveillance.

Most maternal deaths occurred in the early postpartum period (up to 42 days after delivery), accounting for 46.96% of the total. This indicates that the immediate postpartum period continues to be the most critical for maternal health, requiring more effective monitoring and continuous care actions in the postpartum period, including after hospital discharge. Deaths during pregnancy, childbirth or abortion are also significant (28.76%), reinforcing the need to improve prenatal care and the obstetric emergency and urgency network. Late deaths (43 days to less than 1 year) accounted for 3.38% of cases, showing that extended postpartum surveillance is still insufficient, especially for indirect or chronic conditions that worsen in the postpartum period. Cases with an inconsistent (4.83%) or unreported (14.13%) period indicate relevant failures in reporting, which impact classification and hinder the formulation of evidence-based public policies.

The vast majority of maternal deaths (91.13%) occurred in hospitals, indicating that most deaths occur during hospitalization, probably in obstetric reference units or emergency rooms. A small proportion of deaths occurred in other health facilities (2.16%), which may include clinics, basic health units or outpatient clinics. A significant percentage of deaths occurred at home (3.70%) or on public roads (1.28%), which represents critical situations of access or late care and may indicate serious failures in the care network. The categories “other” (1.58%) and “unknown” (0.12%) are small, but reinforce the need to improve the quality and completeness of information.

More than half of maternal deaths (56.51%) were investigated with a duly completed summary form. A significant percentage (8.67%) of deaths were investigated but without a summary form, indicating incomplete documentation. Approximately 10.96% of deaths were not investigated, which reveals weaknesses in the investigation routine and underreporting. The “Not applicable” category (23.85%) may include cases that do not require formal investigation, such as deaths already duly classified or those that occurred outside the gestational period.

Indigenous women had more than twice the risk of maternal death compared to white women (RR = 2.29; 95%CI 2.10–2.48), black and brown women had a 39% higher risk (RR = 1.39) than white women, and yellow women had a similar risk to white women (RR = 1.00). Multinomial logistic regression with the dependent variable type of death (direct, indirect, unspecified); and Poisson regression to estimate adjusted RR of maternal mortality according to skin color, education, region and type of death, presented below in Table 3. Table 3, Table 4, Table 5 and Table 6 include maternal mortality ratios per 100,000 live births, relative risks (RR), and 95% confidence intervals (CI). All values refer to Brazil between 2000 and 2023 based on SIM and SINASC data. Decimal points were standardized, and explanatory footnotes were added to clarify the meaning of each metric.

Table 4 illustrates the stratification of maternal deaths according to key sociodemographic and clinical variables used in the regression models. The distribution highlights that most deaths among Black and Brown women occurred in the Northeast region and were predominantly classified as direct obstetric causes, often among women with low levels of education. Conversely, White women were concentrated in the Southeast region and generally had higher educational attainment. This stratified view reinforces the intersection between race, education, and region as major determinants of maternal mortality in Brazil and provides the analytical basis for the multivariate models presented in subsequent sections.

Indigenous and black skin color, low education level and residence in the North and Northeast regions were independent factors associated with an increased risk of direct maternal death. The Poisson model reinforced that the adjusted relative risk for maternal mortality was more than double for indigenous people compared to the white group (Table 5).

Indigenous women had more than twice the adjusted risk of maternal mortality compared to white women, the North and Northeast regions had a 50–80% higher risk compared to the Southeast, and low education (none or up to 3 years) was strongly associated with increased risk. Indirect and unspecified maternal death were associated with a lower relative risk than direct death (in relation to immediate risk), and extreme ages (<20 and ≥40 years) increased the risk in relation to the 20–29 age group. Infectious and parasitic diseases increased the risk by 45% in relation to direct obstetric causes, HIV increased the risk by 70%. Indirect and unspecified deaths had a lower risk than direct deaths, deaths occurring outside the hospital, especially at home or in public spaces, had a higher relative risk, and uninvestigated deaths presented an increased risk, possibly due to under-reporting or delayed diagnosis (Table 6).

The racial disparities observed in maternal mortality ratios should be understood as indicators of structural and systemic inequities, rather than as a direct measurement of obstetric racism. The classification of skin color in the SIM and SINASC databases may not fully capture Brazil’s racial and ethnic diversity, particularly within the “brown” category, which includes socially and economically heterogeneous groups. Furthermore, the regional gradient identified in maternal deaths underscores the intersection between race, socioeconomic status, and territorial disparities in healthcare access and quality.

These findings can also be interpreted through a syndemic framework, in which social inequalities, racialized structures, and health system inequities interact to amplify the risk of maternal mortality. The racial categories used (Black and Brown) follow the official IBGE classification; however, it is recognized that these terms have sociopolitical limitations and may not fully represent the complexity of racial identity and experience in the Brazilian context.

## 4. Discussion

The results of this study highlight striking inequalities in maternal mortality in Brazil, strongly associated with sociodemographic, clinical, and contextual factors. Multivariate analysis revealed that indigenous women had the highest adjusted risk of maternal mortality compared to white women (adjRR: 2.9; 95% CI: 2.07–2.53), followed by black women (adjRR: 1.39) and brown women (adjRR: 1.36), indicating a persistent pattern of institutionalized structural racism. These racial inequalities were amplified by adverse socioeconomic conditions, such as low education, especially among women with no or up to three years of schooling, whose adjusted relative risks reached 2.14 (95% CI: 1.96–2.34).

These findings are consistent with broader national analyses that have documented disproportionately high maternal mortality rates among Indigenous women across Brazil, underscoring the compounded effects of geographic isolation, systemic neglect, and culturally inappropriate care within the health system [14]. In parallel, maternal mortality among Black women in Brazil is twice as high as that observed among white women, further emphasizing the significance of obstetric racism as a deeply rooted structural issue in Brazilian society [15].

The concept of obstetric racism offers a critical framework for understanding these enduring disparities. Introduced by Davis [16], obstetric racism is defined as the intersection of obstetric violence and medical racism. It acknowledges that, just as obstetric violence is rooted in gender-based power asymmetries, obstetric racism emerges at the nexus of race and gender [16]. This perspective reveals how institutional violence and structural racism intersect to adversely affect the reproductive health of Black women, placing both mothers and their newborns at elevated risk of poor outcomes [17].

It is important to note that the present study does not directly measure obstetric racism, as no individual-level data on discriminatory practices or subjective experiences were available. Instead, the interpretation of racial inequalities in maternal mortality as manifestations of structural and obstetric racism derives from the consistent statistical pattern of higher adjusted risks among black and indigenous women, independent of education, region, and other socioeconomic variables. These findings, interpreted alongside extensive qualitative and epidemiological literature, indicate that racism operates as a structural determinant of health, influencing both the distribution of social resources and the organization of obstetric care [16,17,18,19].

Recent studies conducted in Brazil have documented multiple manifestations of obstetric racism that disproportionately affect Black women. These include limited access to timely and adequate prenatal care, increased likelihood of delays or outright denial of medical attention, and a higher prevalence of invasive procedures performed without informed consent or sufficient pain management. Moreover, Black women’s health concerns are frequently underestimated or dismissed by healthcare providers, contributing to avoidable complications and maternal deaths [18,19]. These patterns of neglect and mistreatment reflect systemic failures that reinforce racialized hierarchies in healthcare delivery and underscore the urgent need for structural reforms to ensure equitable maternal health outcomes.

A critical aspect of this study concerns the use of the variable “race”, which, although central to the analysis of inequalities, presents inherent conceptual and methodological limitations. In the Brazilian context, this variable is self-identified and classified according to categories established by the IBGE: White, Black, Brown, Indigenous and Asian. These categories, while useful for monitoring social inequities, are sociopolitical constructs rather than biological markers. They reflect historical processes of colonization, racialization, and social hierarchy that shape access to rights and health outcomes [20,21,22].

It is important to acknowledge that the variable “race” alone does not fully capture the complex dynamics of racism nor the intersectional effects of gender, class, and territory. In this sense, racial disparities in maternal mortality should be interpreted as part of a broader system of structural and territorial inequalities, rather than as isolated demographic differences. Complementary approaches—such as qualitative studies exploring women’s experiences with discrimination, or multilevel analyses incorporating indicators of social deprivation and geographic segregation, are necessary to deepen the understanding of how racism operates within the health system [23,24,25].

Disparities were also strongly evident in the territorial division, with the North and Northeast regions presenting significantly higher risks of maternal mortality compared to the Southeast, with RRaj of 1.82 and 1.52, respectively. From a clinical point of view, direct obstetric causes remained the main determinant of maternal mortality, but the occurrence of deaths due to infectious and parasitic diseases (RRaj: 1.45) and HIV (RRaj: 1.70) drew attention, revealing weaknesses in the integration of obstetric care with clinical care for chronic and infectious diseases. Deaths recorded as “unspecified” or “not investigated” presented a high risk (RRaj: 1.30). Another relevant finding was the place where the deaths occurred. Women who died outside the hospital environment, especially at home (RRaj: 1.85) or in public spaces (RRaj: 2.30), had a significantly higher risk.

It is important to note, however, that the risk of maternal death increases when home births occur due to a lack of infrastructure and timely access to health services, which is completely different from planned home births. A recent systematic review with meta-analysis pointed out that homebirth is as safe as hospital birth for women who are low risk and attended by professional midwives who, in turn, are well networked into a responsive health system [20]. Homebirths can be less safe for the baby when women with significant risk factors choose it, or when they give birth without regulated health providers in attendance [21].

The educational levels of the women in the sample were low, with only 9.11% having completed more than 11 years of schooling. This suggests that a few women have a university education. Recent research has demonstrated that being a Black woman and not attending school are significant risk factors for maternal mortality in Brazil. The mortality rate of eclampsia was 5,8 times higher among women without any level of education (OR = 5.83; 95% CI: 4.82–7.06) than among those who had completed high school or university education, according to data collected between 2000 and 2021 [22]. Moreover, the risk was 4.7 times greater for black women than for white women (OR = 4.67; 95% CI: 4.18–5.22) [22]. Additional research has demonstrated that the maternal mortality rate among black women is significantly higher, underscoring that these disparities are not solely the result of socioeconomic factors but also of structural racism in obstetric care [23,24].

An exploration of the factors that contribute to racial disparities in maternal morbidity and mortality among black women in Brazil calls for public health, healthcare system, and community-engaged approaches to achieve equity in maternal health outcomes. Racial disparities continue to pose a significant obstacle to maternal and child health in Brazil, with adverse outcomes disproportionately impacting black and Indigenous women and children [25,26]. The historical legacy of slavery and colonialism has resulted in profound consequences for black and Indigenous communities in Brazil, influencing their living conditions, civil rights, and access to essential services [27]. Racism from a systemic viewpoint encompasses all its forms and processes that generate and perpetuate racial inequalities. Extensive documentation exists regarding the racialized disparities in socioeconomic conditions, healthcare access, and health outcomes within the Brazilian population [28,29,30]. Even with the implementation of policies such as the National Policy of Integral Health for the Black Population and the National Policy of Attention to the Health of Indigenous Populations, these inequalities seem to endure [27]. This persistent legacy increases the susceptibility of systematically discriminated populations to health problems, including excess maternal mortality in Brazil [31].

An additional consideration concerns the analytical inclusion of region of residence, which was not explicitly discussed in the initial theoretical framework but emerged as a relevant determinant in the results. From a syndemic and structural perspective, a geographic region operates not merely as a demographic or administrative variable, but as a proxy for structural and territorial inequities that shape access to healthcare and the distribution of social resources. Regional disparities in Brazil reflect historical patterns of development, infrastructure concentration, and uneven implementation of health policies, particularly between the North and Northeast regions, compared to the more affluent South and Southeast [26,27,28].

There is a pressing need to tackle the social determinants contributing to racial disparities in maternal morbidity and mortality by examining how structural racism influences access to essential factors such as quality healthcare (for instance, the impact of structural racism and historical abuses on health-seeking behaviors and trust in the healthcare system), education, income, employment, and nutritious food. Structural racism impacts health through its historical and ongoing effects on the quality of, and equitable access to, crucial social and environmental determinants of health [32].

For example, the practice of redlining has prevented communities of color from obtaining residential mortgages, thereby limiting their access to public transportation, supermarkets, and healthcare, which has exacerbated residential segregation in the United States [33,34,35]. Consequently, in U.S. communities affected by segregation, black individuals and other racial and ethnic minority groups are more likely to reside in neighborhoods characterized by higher poverty levels; to experience diminished access to employment, credit, housing, education, transportation, nutritional, and healthcare resources; and to inhabit health-compromising environments, in contrast to the white population [33,36]. Furthermore, systemic racism obstructs access to essential healthcare services, including reproductive and sexual health services [37]. Therefore, it is imperative to confront these structural barriers and recognize their contribution to racially disparate maternal health outcomes.

The intricate connections between racism and disparities in maternal health are both complex and multifaceted, illustrating the widespread influence of systemic racism on numerous facets of healthcare. Discrimination and bias within healthcare settings can result in unequal access to high-quality prenatal care and maternal services for racial and ethnic minority populations. Moreover, socioeconomic elements shaped by systemic racism, including income disparity and neighborhood segregation, play a significant role in influencing disparities in maternal health outcomes. Chronic stress stemming from experiences of racism may also adversely impact maternal health, potentially resulting in preterm deliveries and low birth weights. In addition, the insufficient representation of minority groups in healthcare decision-making and policy development can sustain these disparities by neglecting to meet the unique needs of these communities. To effectively tackle maternal health inequalities, it is essential to identify and dismantle the structural and institutional obstacles rooted in racism that affect maternal care and outcomes [38].

Barriers to achieving equity in maternal health outcomes could be addressed by targeting the underlying social determinants that fuel the rates of black maternal morbidity and mortality and by incorporating policy and educational modifications to the healthcare system and industries that supply the healthcare system. Relationships in community settings are a contributing factor to maternal health outcomes for black women. This further supports that there is a correlation between health and racial residential segregation. Communities with large black populations tend to be underfunded and lack adequate resources such as stable housing and suitable transportation, which are fundamental causes of poor physical health and further disadvantage the people who live there, which include black pregnant women. This is caused by instances of systematic racism, which cause social and structural determinants of maternal and infant mortality in the USA [32]. Overall, there is a need for anti-racist policies and improved social programs to increase access to reproductive services, improve birth outcomes, and prevent maternal mortality.

The mortality of black women is a critical issue in Brazil and represents a serious public health problem. The concept of reproductive justice can contribute to understanding the factors that increase the mortality of black women, as this concept unfolds in the search for equity in access and guarantee of sexual and reproductive rights. Although the issue of maternal deaths among black women is an expression of racism in reproductive health, it also represents injustice due to the lack of effective and/or quality access to sexual and reproductive rights and adequate maternity care when compared to white women [39,40].

Data that contradict the SDGs in Brazil supported by the UN and its partners, namely, 3.7, which by 2030 has as a premise ensuring universal access to sexual and reproductive health services, 10.3 which guarantees equality and the reduction in inequalities including through the elimination of discriminatory practices. Emblematic case of the Alyne Network that contravenes Law No. 10,237, 12/03/1999, instituted to overcome racial discrimination in the State, which guarantees everyone, without any distinction of race, color and origin, equal opportunity of access to work, education, health, housing, leisure and security [41,42].

Maternal mortality remains one of the principal global public health challenges, reflecting socioeconomic conditions, inequalities, access to quality services, and the effectiveness of health policies. In 2022, the global MMR was estimated at approximately 292 deaths per 100,000 live births, with significant disparities among world regions. High-income countries report rates below 10 deaths per 100,000, while low- and middle-income nations, particularly in Sub-Saharan Africa and South Asia, exceed 500 deaths per 100,000 live births. Sub-Saharan Africa accounts for around 70% of global maternal deaths, with lifetime risk reaching as high as 2% for women of reproductive age [43,44,45]. Improving maternal health depends on the integration of evidence-based policies and contextually oriented actions. While Ward et al. (2024) provide a macro model for global categorization and planning, Oluwole et al. (2025) bring local empirical evidence on inequalities in access to and use of services, demonstrating how social and economic determinants shape maternal outcomes [46,47].

Maternal mortality rates in Brazil, currently around 62 deaths per 100,000 live births, indicate significant progress compared to past decades, when rates were roughly double. However, this figure still exceeds the United Nations target of a maximum of 35 deaths per 100,000 live births for developing countries under the SDGs by 2030. Compared to other developing nations, Brazil has managed a continuous reduction in maternal mortality, although significant regional variation persists, particularly in the North and Northeast regions, which face more severe socioeconomic and structural challenges. In similar middle-income countries, maternal mortality frequently surpasses international targets, reflecting inequalities in access to, and quality of, obstetric and prenatal care. Factors such as high elective cesarean rates also contribute to increased risks in Brazil, as studies link cesareans to higher morbidity and maternal mortality due to infectious, hemorrhagic, and anesthetic complications. Despite initiatives by the Ministry of Health to encourage vaginal birth and strategies to promote qualified assistance, the country encounters difficulties in reaching ideal rates, influenced by cultural, ethical, and socioeconomic aspects that affect reproductive choices and access to proper obstetric care [48,49,50].

Therefore, while the Brazilian context shows improvements, it remains aligned with challenges common to many developing countries, where social inequalities and insufficient health infrastructure hinder rapid reductions in maternal deaths. This comparison highlights the need to strengthen public policies, expand the quality of primary and specialized care, and promote equity-focused actions so that Brazil can move closer to rates observed in middle-high income countries and meet its global commitments related to maternal health. Given this scenario, reducing maternal mortality is a multidimensional challenge requiring integrated actions. A comprehensive approach is essential, not only clinical improvements, but also interventions aimed at enhancing the quality of primary and specialized care, equity, women’s empowerment, and social development, alongside stronger public health policies as central strategies to tackle this serious global and Brazilian problem [51,52].

The incorporation of the theoretical framework of syndemics significantly enriches the interpretation of the findings of this study, as it shifts the explanation from isolated adverse events to an understanding of the complex interactions between biological, social, and structural processes that increase the risk of maternal death. The syndemic approach is based on the principle that health problems do not occur in isolation, but tend to cluster and interact in vulnerable populations under the influence of adverse social determinants, poverty, institutional racism, gender violence, environmental precariousness, and territorial exclusion that reinforce and potentiate their harmful effects [53].

Applied to maternal mortality, the syndemic perspective guides an interpretation in which racial, social, and territorial inequalities are not merely covariates, but active components of an interactive process that amplifies risks: for example, the confluence of low education, housing in territories with insufficient health services, and obstetric discrimination practices can simultaneously increase exposure to infectious diseases, delays in recognizing risk signs, and barriers to accessing emergency care, effects that, together, increase the probability of fatal outcomes. Thus, socioeconomic and institutional factors function as risk multipliers (syndemic drivers), not just as independent antecedents [54].

Merrill Singer has substantially expanded the conceptual framework of syndemics since 2020, advancing analyses that integrate structural vulnerabilities, racial inequalities, and interactive effects between social conditions and health problems. In his most recent work [55,56,57], Singer highlights that syndemics are not merely the sum of diseases, but systems of co-occurrence shaped by political and economic forces that produce differentiated harm according to race, territory, and social position. In post-pandemic publications, Singer emphasizes how health crises, such as COVID-19, amplify existing inequalities, especially among racialized and poor populations, by producing “pathogenic couplings” between infections, institutional discrimination, poverty, state violence, and the deterioration of care networks [55,57].

In this sense, the contemporary concept of syndemic reinforces that populations already exposed to structural racism, such as Black and Indigenous women in Brazil, face multiplied risks of serious outcomes, including maternal mortality, due to synergistic interactions between clinical conditions, precarious health services, overload of infectious diseases, and institutional barriers to timely access to care. The racial inequalities found are not isolated variables, but expressions of syndemic processes that deepen historical vulnerabilities and sustain persistent patterns of increased risk of maternal death [56,57].

Recent literature on maternal health and racial disparities reinforces that population shocks (e.g., the COVID-19 pandemic) acted as catalysts for syndemics, exposing how pre-existing vulnerabilities made certain populations, particularly Black and Indigenous women, more susceptible to adverse outcomes. This picture shows that strictly biomedical interventions (improvement of hospital routines, clinical protocols) are necessary but insufficient: to significantly reduce maternal mortality, it is necessary to intervene in the structural determinants that underpin syndemic vulnerability, poverty reduction policies, anti-racist actions in the health sector, expansion of the obstetric network in peripheral territories, and qualified investigation of deaths [58,59].

From a methodological and policymaking perspective, the syndemic approach also implies three practical consequences for research on maternal mortality: (1) prioritizing measures capable of capturing interactions between factors (and not just additive effects), (2) integrating clinical and social data, for example, links between death records and territorial indicators of deprivation, and (3) promoting multisectoral and territorialized interventions, evaluated by designs that consider combined effects and synergies between actions [54,58]. In short, the syndemic framework offers an interpretative and programmatic structure to explain why maternal mortality is concentrated among black women and in less favored regions, not as a sum of isolated factors, but as a result of multiplicative interactions between biological conditions, health services, and social and racial inequalities [53].

In addition to addressing the structural, racial and territorial determinants of maternal mortality, it is also essential to consider support mechanisms for families who experience the death of a mother or other severe maternal outcome. The trauma of losing a partner, mother or child in the perinatal or maternal period carries profound emotional, social and economic consequences for surviving family members, including children, partners and extended households.

Healthcare systems and maternal health programs should therefore incorporate post-event psychosocial support, such as: (1) early and compassionate communication of the event; (2) structured grief counseling and bereavement support groups; (3) follow-up by community health teams to ensure continuity of care for surviving family members; and (4) recognition of the loss through memorials or similar practices that affirm the family’s experience. For example, evidence from Brazil shows that the implementation of guidelines for perinatal bereavement improved mental health outcomes among affected families [59].

Despite the methodological robustness and national scope of the database used, this study has some important limitations. The first concerns the quality and completeness of the information recorded in the SIM, especially regarding the variable “race”, which still suffers from underreporting and inconsistent completion in some regions of the country. This may introduce differential classification bias and impact the accuracy of the estimates. In addition, the classification of causes of death, particularly in regions with poor health services, may suffer from underdiagnosis or incorrect coding, resulting in underestimation of maternal deaths or their classification as non-obstetric causes. Another limitation is the retrospective and observational nature of the study, which prevents the inference of direct causality between the variables analyzed. Although the statistical models controlled for sociodemographic and regional factors, it was not possible to include individual clinical variables, such as number of prenatal consultations, comorbidities or type of delivery, as they were not available in the database used. In addition, the absence of qualitative data prevents an in-depth understanding of the subjective experiences of black women in the care pathway.

Despite its limitations, the study presents important contributions to the field of public health and racial equity. It is one of the most comprehensive analyses of maternal mortality in Brazil focusing on racial inequalities, covering an extensive period of 24 years and using advanced statistical techniques to adjust for confounding factors. The main contribution lies in the empirical evidence that black and indigenous women are systematically more exposed to the risk of maternal death, regardless of their level of education and region, which reinforces the urgency of intersectional public policies. By articulating quantitative data with the milestones of the Rede Alyne and the commitments assumed by Brazil in the SDGs, the study also provides relevant support for managers, researchers and social movements engaged in reducing maternal mortality with social and racial justice.

## 5. Conclusions

The findings of this study demonstrate that maternal mortality in Brazil remains a critical public health issue, profoundly shaped by racial, social, and territorial inequalities. Black and Indigenous women continue to face disproportionately higher risks of death during pregnancy, childbirth, and the postpartum period, reflecting the intersection of structural social determinants including poverty, low educational attainment, territorial exclusion, and institutional racism, with persistent shortcomings in the organization and quality of obstetric care.

Interpreting these results through the lens of the syndemic theoretical framework provides a more comprehensive understanding of maternal mortality, emphasizing that these outcomes do not result from isolated biological or clinical factors, but rather from the synergistic interactions among social, economic, cultural, and environmental determinants that mutually reinforce vulnerability. This perspective deepens the explanatory power of the analysis by showing how overlapping disadvantages, such as living in under-resourced regions, experiencing racial discrimination in healthcare settings, and facing systemic barriers to timely care, create a context in which maternal risks are intensified and health inequities perpetuated.

Accordingly, public policies aimed at reducing maternal mortality must adopt intersectoral and anti-racist strategies that integrate actions across health, education, housing, and social protection systems. Strengthening Brazil’s Unified Health System based on principles of equity, territoriality, and cultural competence is essential, alongside improving surveillance and the qualified investigation of maternal deaths, particularly in socioeconomically and geographically vulnerable contexts. Moreover, incorporating interdisciplinary approaches and the syndemic perspective into research and policymaking can guide more effective interventions capable of addressing the root causes of health inequities and reducing preventable maternal deaths. Ultimately, reducing maternal mortality requires not only technical or clinical advances but also structural and cultural transformations—recognizing racism as a determinant of health, safeguarding reproductive rights, and promoting care grounded in social justice and respect for diversity. In this sense, the present study contributes to advancing an agenda of reproductive justice and health equity, essential for Brazil to meet its commitments under the SDGs and to ensure every woman’s right to life and safe motherhood.

## Figures and Tables

**Table 1:** Univariate descriptive analysis of sociodemographic characteristics of women with maternal death in Brazil (2000–2020).

	Absolute Frequency (*n*)	Relative Frequency (%)
**Age range**		
10 to 14 years old	346.00	0.85%
15 to 19 years old	5.10	12.48%
20 to 29 years old	16.38	40.05%
30 to 39 years old	15.55	38.00%
40 to 49 years old	3.42	8.36%
50 to 59 years old	85.00	0.21%
70 to 79 years old	1.00	0%
Age ignored	23.00	0.06%
**Race**		
White	13.65	33.37%
Black	4.45	10.87%
Brown	19.77	48.33%
Yellow	123.00	0.30%
Indigenous	566.00	1.38%
Ignored	2.35	5.75%
**Region**		
North	5.15	12.58%
North East	13.75	33.61%
Southeast	14.13	34.55%
South	4.55	11.14%
Midwest	3.33	8.13%
**Marital status**		
Single	20.58	50.30%
Married	12.34	30.17%
Widow	307.00	0.75%
Legally separated	696.00	1.70%
Other	3.82	9.34%
Ignored	3.17	7.74%
**Education**		
None	1.53	3.75%
1 to 3 years	4.66	11.39%
4 to 7 years	9.54	23.33%
8 to 11 years old	12.15	29.69%
12 years and over	3.72	9.11%
9 to 11 years old	3.00	0.01%
Ignored	9.30	22.74%
**Total**	40,907	100%

**Source:** Prepared by the author. Brazil, 2025.

**Table 2:** Profile of maternal deaths according to the ICD-10 classification and associated variables.

	Absolute Frequency (*n*)	Relative Frequency (%)
**ICD-10 Chapter**		
I. Some infectious and parasitic diseases	844.00	2.06%
II. Neoplasms (tumors)	10.00	0.02%
IV. Endocrine, nutritional and metabolic diseases	1.00	0%
V. Mental and behavioral disorders	36.00	0.09%
Subtotal indirect causes	891.00	2.18%
**ICD-10 Group**		
Human immunodeficiency virus [HIV] disease (B20–B24)	841.00	2.06%
Other bacterial diseases	3.00	0.01%
Neoplasms of uncertain or unknown behavior	10.00	0.02%
Disorders of other endocrine glands	1.00	0%
Behavioral syndromes associated with physiological dysfunctions and physical factors (F50–F59)	36.00	0.09%
Pregnancy ending in abortion (O00–O08)	3.19	7.81%
Edema, proteinuria and hypertensive disorders in pregnancy, childbirth and the puerperium (O10–O16)	8.75	21.39%
Other maternal disorders predominantly related to pregnancy (O20–O29)	1.55	3.80%
Care of the mother for reasons related to the fetus, amniotic cavity and problems during delivery (O30–O48)	2.60	6.36%
Complications of labor and delivery (O60–O75)	6.23	15.23%
Childbirth (O80–O84)	1.00	0%
Complications related predominantly to the puerperium (O85–O92)	5.42	13.26%
Other obstetric conditions, not elsewhere classified (NCOP–O95–O99)	12.26	29.98%
**Type of maternal death**		
Direct obstetric maternal death	26.96	65.93%
Indirect obstetric maternal death	12.66	30.96%
Unspecified obstetric maternal death	1.28	3.13%
**Time of death**		
During pregnancy, childbirth or abortion	11.76	28.76%
During the puerperium, up to 42 days	19.20	46.96%
During the puerperium, from 43 days to less than 1 year	1.38	3.38%
Not in pregnancy or postpartum	803.00	1.96%
Inconsistent reported period	1.98	4.83%
Not informed or ignored	5.78	14.13%
**Place of occurrence**		
Hospital	37.29	91.13%
Other health facility	882.00	2.16%
Domicile	1.51	3.70%
Public road	525.00	1.28%
Others	648.00	1.58%
Ignored	51.00	0.12%
**Investigation status**		
Death investigated, with summary record	23.12	56.51%
Death investigated, without summary record	3.55	8.67%
Death not investigated	4.48	10.96%
Not applicable	9.75	23.85%

**Source:** Prepared by the author. Brazil, 2025.

**Table 3:** Distribution of maternal deaths, live births, maternal mortality rates and risk ratio by skin color.

Race	Deaths	Live Births	Rate (per 100,000)	RR vs. White	Lower 95% CI	Upper 95% CI
White	13.65	884,000	1.544	—	—	—
Black	4.45	207,000	2.146	1.39	1.35	1.44
Yellow	123.00	8000	1.537	1.00	0.84	1.19
Brown	19.77	920,000	2.148	1.39	1.36	1.42
Indigenous	566.00	16,000	3.538	2.29	2.10	2.48

**Source:** Prepared by the author. Brazil, 2025. RR (Relative Risk) was calculated using Poisson regression with the white category as the reference group 95% CI = 95% confidence interval rates are expressed per 100,000 live births based on data from SIM and SINASC.

**Table 4:** Stratification of maternal deaths by sociodemographic and clinical variables.

Race	Education	Region	Direct	Indirect	Not Specified	Total
White	8–11 years	Southeast	3.000	1.200	300	4.500
White	1–3 years	North East	1.500	800	100	2.400
Black	1–3 years	North East	1.200	600	80	1.880
Brown	4–7 years	North East	2.500	1.000	150	3.650
Indigenous	None	North	400	150	50	600

**Source:** Prepared by the author. Brazil, 2025.

**Table 5:** Poisson model for adjusted maternal mortality and adjusted estimate.

Variable	Category	RR	95% CI	*p*-Value
Race	Black	1.35	1.18–1.53	<0.001
Race	Brown	1.30	1.15–1.46	<0.001
Race	Indigenous	2.15	1.85–2.50	<0.001
Education	1–3 years	1.50	1.33–1.70	<0.001
Education	None	2.00	1.70–2.35	<0.001
Region	North East	1.45	1.30–1.62	<0.001
Region	North	1.80	1.50–2.15	<0.001

**Source:** Prepared by the author. Brazil, 2025. RRadj = adjusted relative risk estimated by Poisson regression with robust variance. Models were adjusted for skin color, education, region, type and place of death, ICD-10 group, and age. 95% CI = 95% confidence interval. *p*-value: the *p*-values reported in the table correspond to the Wald test for each coefficient in the regression model; *p*-values are two-sided, and statistical significance was set at *p* < 0.05.

**Table 6:** Estimation of adjusted risk ratio (RRaj), clinical and death characteristics for maternal mortality in Brazil (2000–2020).

Variable	Category	RRaj	95% CI	*p*-Value
**Skin Color**	White (reference)	1.00	—	—
	Black	1.39	1.30–1.48	<0.001
	Brown	1.36	1.29–1.43	<0.001
	Yellow	0.98	0.70–1.36	0.911
	Indigenous	2.29	2.07–2.53	<0.001
**Region**	Southeast (reference)	1.00	—	—
	North	1.82	1.68–1.96	<0.001
	North East	1.52	1.45–1.59	<0.001
	South	1.05	0.98–1.13	0.182
	Midwest	1.08	0.99–1.18	0.085
**Education**	8–11 years (reference)	1.00	—	—
	None	2.14	1.96–2.34	<0.001
	1 to 3 years	1.61	1.52–1.71	<0.001
	4 to 7 years	1.28	1.22–1.35	<0.001
	12 years or older	0.89	0.83–0.95	0.001
**Type of Death**	Direct (reference)	1.00	—	—
	Indirect	0.62	0.59–0.66	<0.001
	Not specified	0.55	0.49–0.62	<0.001
**Age**	20–29 years (reference)	1.00	—	—
	<20 years	1.15	1.07–1.24	<0.001
	30–39 years old	1.22	1.15–1.29	<0.001
	≥40 years	1.58	1.42–1.75	<0.001
**Chapter ICD-10**	Pregnancy, childbirth and puerperium (reference)	1.00	—	—
	Infectious and parasitic diseases	1.45	1.30–1.62	<0.001
	Neoplasms	0.85	0.60–1.20	0.349
	Endocrine, nutritional and metabolic diseases	1.10	0.75–1.62	0.62
	Mental and behavioral disorders	1.15	0.95–1.39	0.14
	Other obstetric causes (NCOP)	1.25	1.10–1.42	0.001
**ICD-10 Group**	Complications of pregnancy and childbirth (reference)	1.00	—	—
	Other bacterial diseases	1.40	0.85–2.32	0.185
	HIV	1.70	1.50–1.92	<0.001
	Neoplasms of uncertain behavior	0.90	0.55–1.48	0.67
	Endocrine gland disorders	1.05	0.55–2.02	0.89
	Associated behavioral syndromes	1.20	0.85–1.68	0.30
**Type of death**	Direct obstetric death (reference)	1.00	—	—
	Indirect obstetric death	0.75	0.72–0.79	<0.001
	Unspecified obstetric death	0.65	0.58–0.73	<0.001
**Place of death**	Hospital (reference)	1.00	—	—
	Other health facility	1.10	1.00–1.20	0.05
	Domicile	1.85	1.65–2.08	<0.001
	Public road	2.30	1.80–2.95	<0.001
	Others	1.15	0.90–1.47	0.25
**Death investigation**	Investigated with summary sheet (reference)	1.00	—	—
	Investigated without summary record	1.12	1.04–1.21	0.003
	Not investigated	1.30	1.20–1.42	<0.001
	Not applicable	0.95	0.89–1.02	0.14

**Source:** Prepared by the author. Brazil, 2025. RRadj = adjusted relative risk estimated by Poisson regression with robust variance. Models were adjusted for skin color, education, region, type and place of death, ICD-10 group, and age. 95% CI = 95% confidence interval. *p*-value: the *p*-values reported in the table correspond to the Wald test for each coefficient in the regression model; *p*-values are two-sided, and statistical significance was set at *p* < 0.05.

## Data Availability

The data presented in this study are available at Sistema de Informação sobre Mortalidade—SIM (https://opendatasus.saude.gov.br/dataset/sim, accessed on 10 July 2025; http://tabnet.datasus.gov.br/cgi/tabcgi.exe?sim/cnv/mat10uf.def, accessed on 10 July 2025) and Sistema de Informação sobre Nascidos Vivos—SINASC (https://opendatasus.saude.gov.br/dataset/sistema-de-informacao-sobre-nascidos-vivos-sinasc, accessed on 10 July 2025; http://tabnet.datasus.gov.br/cgi/tabcgi.exe?sinasc/cnv/nvuf.def, accessed on 10 July 2025).

## Abbreviations

CEDAW	Committee on the Elimination of Discrimination against Women
DATASUS	Departamento de Informação e Informática do Sistema Único de Saúde
IBGE	Instituto Brasileiro de Geografia e Estatística
ICU	Intensive Care Unit
ICD-10	International Classification of Diseases
MMR	Maternal mortality ratio
OR	Odds ratio
RR	Risk ratio
SDGs	Sustainable Development Goals
SIM	Sistema de Informações sobre Mortalidade
SINASC	Sistema de Informações sobre Nascidos Vivos
SUS	Sistema Único de Saúde
TABNET	Tabulador de Dados da Internet
USA	United States of America
95% CI	95% confidence interval
